# Halofuginone- and Chitosan-Coated Amnion Membranes Demonstrate Improved Abdominal Adhesion Prevention

**DOI:** 10.1100/tsw.2010.234

**Published:** 2010-12-14

**Authors:** Scott Washburn, Jamie L. Jennell, Steve J. Hodges

**Affiliations:** ^1^ Department of Obstetrics and Gynecology, Wake Forest University School of Medicine, Winston-Salem, NC 27157, USA; ^2^ Department of Urology, Wake Forest University School of Medicine, Winston-Salem, NC 27157, USA

**Keywords:** halofuginone, chitosan, amnion, abdominal adhesions

## Abstract

Our objective was to determine whether coating the amniotic membrane with halofuginone, a type 1 collagen synthase inhibitor, with or without the hemostasis-inducing substance chitosan, reduced the number and severity of adhesions in the rat uterine horn injury model. Sixty retired breeder Sprague-Dawley rats underwent midline laparotomy and a zone of ischemia was created in the left uterine horn of each animal. Rats were randomized to one of six treatment groups: (1) untreated control, (2) oxidized regenerated cellulose (Interceed®) (ORC), (3) plain amnion, (4) amnion coated on both sides with 0.5% solution of halofuginone (HAH), (5) amnion coated on one side with 0.5% halofuginone and on the other side with chitosan (CAH), or (6) amnion coated on both sides with chitosan (CAC). The zone of ischemia in each left uterine horn was wrapped in each treatment. Rats were sacrificed 2 weeks after laparotomy, and adhesions were counted and scored for severity. Data were analyzed using Chi square and a *p* <0.05 was considered significant. Our results showed that there were no differences in the percentage of animals with adhesions in the untreated, ORC, plain amnion, or CAC groups. No adhesions formed in any animal in the HAH group and only 14% of the animals developed adhesions to the uterine horn in the CAH group (*p* < 0.05). The percentage of animals with moderate and severe adhesions did not differ between untreated controls and the ORC groups, but were significantly reduced in all four of the amnion groups: plain amnion, HAH, CAH, and CAC (*p* < 0.05). Amnion coated with halofuginone alone or in combination with chitosan reduced the percentage of animals with adhesions, as well as the percentage of animals with moderate and severe adhesions compared to untreated controls and the ORC group in the rat uterine horn injury model. Amnion alone or coated with chitosan reduced the percentage of rats with moderate and severe adhesions, but not the percentage of rats with adhesions of any type compared to both untreated controls and the ORC group in the rat uterine horn injury model.

## BACKGROUND

Postsurgical adhesions are a common cause of surgical morbidity. Adhesions distort normal anatomic relationships, which can cause pain and necessitate additional corrective surgeries. Unfortunately, additional surgeries can exacerbate adhesions and lead to additional and more severe complications. Many strategies have been attempted to prevent adhesions resulting from intraoperative tissue manipulation and injury, and postoperative oozing. Prevention strategies vary from perioperative instillation of colloidal solutions, to both biologic and synthetic barriers to systemic steroids. None are perfect remedies.

The human amnion has been demonstrated to effectively prevent adhesions in animal models[1]. The preparation and storage of amniotic membranes for use in perioperative adhesion prevention has previously been cumbersome and inordinately expensive. Recently, an amnion product has been developed that can be stored at room temperature for prolonged periods of time prior to use[2]. The preparation has proven extremely effective in preventing scleral scarring following pterygm removal[3].

Halofuginone, a potent inhibitor of type 1 collagen synthesis, prevents adhesions in a rat model in a dose-dependent manner[4]. The investigators treated the rats with either oral or intraperitoneal injections of halofuginone for approximately 1 week prior to surgery and for 3 weeks postoperatively, when the animals were necropsied.

Chitosan, poly-N-acetyl glucosamine, is a recent and highly effective component of a bandage preparation for treating trauma-induced hemorrhage in the field. Chitosan-impregnated bandages have been used extensively in the Iraq and Afghanistan theaters. Chitosan does not induce tissue injury or adhere to the underlying tissue, and has been shown to have bacteriostatic properties. Chitosan has also been shown to effectively reduce adhesion formation in rat models of tissue abrasion, tissue ischemia, and tissue infection[5]. One possible mechanism of action of chitosan's adhesion prevention is its ability to prevent fibroblast and macrophage adherence to the injury site[6].

We tested the effectiveness of amnion coated with halofuginone on both sides, or halofuginone on one side and chitosan on the opposite side, in preventing peritoneal adhesions and reducing the severity of adhesions that formed in the rat uterine horn injury model.

## MATERIALS AND METHODS

### Animal Procedures

After approval by the Wake Forest University Health Sciences Animal Care and Use Committee, 60 retired breeder female Sprague Dawley rats (Charles River Labs, Charles River, NJ) were randomly assigned to one of six treatment groups: (1) untreated control, (2) oxidized regenerated cellulose (ORC) barrier control (Interceed®, Johnson and Johnson, Cincinnati, OH), (3) plain amnion, (4) halofuginone/amnion/halofuginone (HAH); (5) chitosan/amnion/halofuginone (CAH), or (6) chitosan/amnion/chitosan (CAC). The animals were kept in single-cage housing with an ambient room temperature of approximately 72°F, with 12-h light and dark cycles and ad lib access to pelleted rat food and drip water. After assignment to treatment groups, each animal was anesthetized with 1.5–2% isoflurane in oxygen general anesthesia, with spontaneous breathing during the procedure. Following successful anesthesia and utilizing clean surgical technique, each rat's abdominal wall was cleaned with isopropyl alcohol and a 3- to 4-cm midline, abdominal incision was performed. The left uterine horn of each uterus was elevated into the incision. A zone of ischemia was created by crushing each left horn with a hemostat for approximately 30 sec. The longitudinal vascular bundle of the horn was not compromised. After the uterine horn injury procedure was completed, the injured uterine horn was wrapped in the adhesion barrier of the animal's assigned treatment group (the injured uterine horn of the untreated control animals was not wrapped). The animals were sacrificed 2 weeks postoperatively using pentobarbital and necropsied. The surgeons did not necropsy the animals they had operated on initially. At necropsy, the animals were assessed for the presence or absence of adhesions involving the injured uterine horn using a modification of the method describe by Leach et al.[7]. The animals were evaluated as to whether they had adhesion formation or not. The degree of adhesion formation was evaluated as being minimal to mild (adhesions had no resistance to separation), or moderate to severe (separation required moderate force to sharp dissection).

## Barrier Preparations: Collagen Substrate and Barrier Preparation

Patients completed Wake Forest University Health Sciences and Forsyth Medical Center Institutional Review Board–approved informed consent during their routine presurgical anesthesia visit for elective cesarean section at term. Following delivery of the infant and placenta, the amniotic membrane was aseptically dissected from each placenta. The chorion was then discarded and the remaining collagen sheet was chemically and mechanically cleaned with normal saline and antibiotics (sterile normal saline with 0.02% streptomycin sulfate and 0.015% gentamicin sulfate [Biologos, Montgomery, IL]).

The "plain amnion sheets" (Amnion) were constructed by utilizing approximately 200 $cm^2$ of the collagen sheet, which was then dried in a biological safety cabinet, cut into 2- × 3-cm rectangles, laid flat in pouches, sealed, labeled, and irradiated.

The HAH sheets were constructed by utilizing approximately 200 $cm^2$ of the collagen sheet and submerging it in the halofuginone gel solution and refrigerated at $4^oC$ for 10 h, followed by drying in a biological safety cabinet. The coated matrix was cut into 2- × 3-cm rectangles, laid flat in pouches, sealed, labeled, and irradiated. The halofuginone coating was made by mixing 1.5 g of sodium alginate (Spectrum Chemical, Gardena, CA) into 75 ml of sterile distilled water (Baxter, Deerfield, IL). The mixture was heated and agitated to form a flowing gel. The gel was cooled to room temperature. Then, 20 ml of gel was mixed with 20-ml halofuginone 0.5-mg/ml solution (Halocur®, Intervet, Intervet Ireland) to form a viscous liquid with uniform color.

The CAH sheets were constructed by utilizing approximately 200 $cm^2$ of the collagen sheet. The chitosan gel mixture was spread in a uniform manner across a dying fixture. A prepared layer of the collagen sheet was placed on top of that, and then a top layer of halofuginone gel was spread upon it. The resulting configuration was placed in a biological safety cabinet and dried, then cut into uniform rectangles, laid flat in pouches, sealed, labeled, and irradiated. Chitosan gel was made by mixing 0.5 g of chitosan (Tokyo Chemical Industry, Tokyo, Japan for TCI America) added to 30-ml acetic acid, 1% aqueous solution (Elecron Microscopy Sciences, Hatfield, PA), heated and agitated to form a gel, then cooled to room temperature.

The CAC sheets were constructed by utilizing approximately 200 $cm^2$ of the collagen sheet. The chitosan gel mixture was spread in a uniform manner across a dying fixture. A prepared layer of the collagen sheet was placed on top of that, and then a top layer of chitosan gel was spread upon it. The resulting configuration was placed in a biological safety cabinet and dried, then cut into uniform rectangles, laid flat in pouches, sealed, labeled, and irradiated.

## Statistical Methods

Prior studies have demonstrated that a number (n) of 10 animals per treatment group is sufficient to determine a significant treatment effect at a level of $p < 0.05$ with 90% power[1,4,5]. The percentage of animals in a treatment group with adhesions to the injured uterine horn and the percentage of animals in a treatment group with adhesions that were scored either 2 or 3 (requiring countertraction or sharp dissection to lyse) were compared to the untreated and ORC controls by Chi square analysis. A $p$ value of <0.05 was considered statistically significant.

## RESULTS

As depicted in Fig. 1a, both the HAH and the CAH barriers prevented adhesions when compared to either untreated or ORC-treated controls. Plain amnion and CAC adhesion barriers did not significantly reduce adhesion formation compared to either untreated or ORC barrier controls. Similarly, Fig. 1b depicts that the percentage of animals with moderate and/or dense adhesions was significantly reduced by both the HAH and CAH barriers compared to untreated and ORC-treated controls. There was no evidence of any residual adhesion barrier in any animal at necropsy.

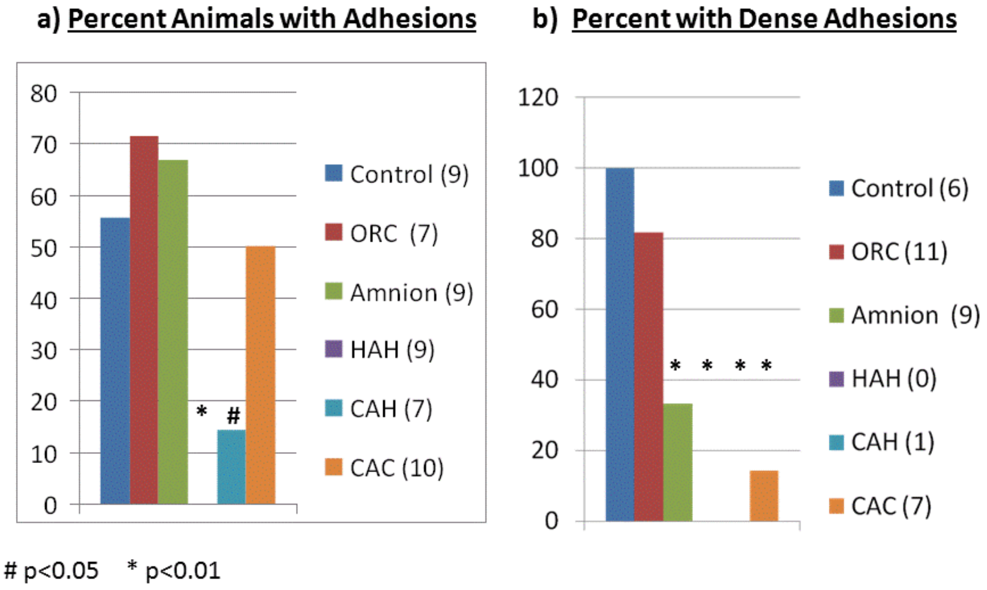

**FIGURE 1.**

## DISCUSSION

Surgical adhesions cause significant morbidity and can result in organ dysfunction and chronic pain syndromes. Repeat surgeries for complications of adhesion-related abnormalities can worsen adhesions and increase adhesion-related morbidity. Preventing adhesion formation and reformation in surgery remains a stubborn surgical problem. Many treatment modalities have met with limited success and include absorbable and nonabsorbable synthetic barriers; absorbable biologic barriers; locally applied, drug-containing gels and systemic steroids; and other anti-inflammatory medicines.

Our experiment tested whether a collagen substrate derived from human amniotic membrane alone or coated with chitosan gel (poly-N-acetyl glucosamine) or halofuginone gel (a collagen synthase inhibitor), or coated with chitosan gel on one side and halofuginone gel on the other, would effectively prevent adhesion formation in the rat uterine horn injury model. We have demonstrated two, multimodal, bioabsorbable adhesion barriers that effectively prevent adhesions and reduce the severity of the adhesions that do form. The two barriers that were effective were constructed with a collagen substrate derived from human amniotic membrane that was coated on both sides with halofuginone gel or was coated on one side with halofuginone gel and the other side with chitosan gel. We believe that the efficacy of those two barriers is a result of the physical barrier of the collagen substrate coupled with inhibition of collagen synthesis by halofuginone, and the possible prevention of fibroblast and macrophage adherence by the chitosan gel.

We were disappointed that the ORC control barrier, plain amnion, and CAC barriers were no more effective at preventing adhesions than the untreated controls. The decreased efficacy of those treatments may be the result of our not apposing or attaching the injured uterine horn to another injured peritoneal surface, since we allowed the injured uterine horn to remain "free floating" in the abdomen. The decreased efficacy of the other treatments may also have been the result of use of "clean" and not "sterile" surgical techniques. The excellent effectiveness of the HAH and CAH treatments at preventing adhesion formation and reducing adhesion severity scores under these conditions is even more remarkable.

While the results of this experiment are robust, future experiments will be needed to elucidate the optimal dose of both chitosan and halofuginone. Additional experiments will be needed to test the efficacy of these barriers for achieving hemostasis, preventing adhesion reformation, and preventing adhesions from a variety of nidus. We believe our multimodal approach to adhesion prevention is promising.

## ACKNOWLEDGMENTS

Animals and related costs were funded through the Frank C. Greiss, MD, Memorial Research Fund of the Department of Obstetrics and Gynecology of the Wake Forest University School of Medicine, Winston-Salem, NC. The amnion adhesion barriers were constructed in the labs of Ocular Systems, Winston-Salem, NC.
